# Interleukin 2-regulated *in vitro *antibody production following a single spinal manipulative treatment in normal subjects

**DOI:** 10.1186/1746-1340-18-26

**Published:** 2010-09-08

**Authors:** Julita A Teodorczyk-Injeyan, Marion McGregor, Richard Ruegg, H Stephen Injeyan

**Affiliations:** 1 Associate Professor, Graduate Education and Research, Canadian Memorial Chiropractic College, 6100 Leslie Street, Toronto, Ontario, M2 H 3J1, Canada; 2 Professor, Undergraduate Education, Canadian Memorial Chiropractic College, Canada; 3 Assistant Professor and Associate Dean of Clinics, Canadian Memorial Chiropractic College, Canada; 4 Professor and Chair, Department of Pathology and Microbiology, Canadian Memorial Chiropractic College, Canada

## Abstract

**Background:**

Our recent investigations have demonstrated that cell cultures from subjects, who received a single spinal manipulative treatment in the upper thoracic spine, show increased capacity for the production of the key immunoregulatory cytokine, interleukin-2. However, it has not been determined if such changes influence the response of the immune effector cells. Thus, the purpose of the present study was to determine whether, in the same subjects, spinal manipulation-related augmentation of the *in vitro *interleukin-2 synthesis is associated with the modulation of interleukin 2-dependent and/or interleukin-2-induced humoral immune response (antibody synthesis).

**Methods:**

A total of seventy-four age and sex-matched healthy asymptomatic subjects were studied. The subjects were assigned randomly to: venipuncture control (n = 22), spinal manipulative treatment without cavitation (n = 25) or spinal manipulative treatment associated with cavitation (n = 27) groups. Heparinized blood samples were obtained from the subjects before (baseline) and then at 20 minutes and 2 hours post-treatment. Immunoglobulin (antibody) synthesis was induced in cultures of peripheral blood mononuclear cells by stimulation with conventional pokeweed mitogen or by application of human recombinant interleukin-2. Determinations of the levels of immunoglobulin G and immunoglobulin M production in culture supernatants were performed by specific immunoassays.

**Results:**

The baseline levels of immunoglobulin synthesis induced by pokeweed mitogen or human recombinant interleukin-2 stimulation were comparable in all groups. No significant changes in the production of pokeweed mitogen-induced immunoglobulins were observed during the post-treatment period in any of the study groups. In contrast, the production of interleukin-2 -induced immunoglobulin G and immunoglobulin M was significantly increased in cultures from subjects treated with spinal manipulation. At 20 min post-manipulation, immunoglobulin G synthesis was significantly elevated in subjects who received manipulation with cavitation, relative to that in cultures from subjects who received manipulation without cavitation and venipuncture alone. At 2 hr post-treatment, immunoglobulin M synthesis was significantly elevated in subjects who received manipulation with cavitation relative to the venipuncture group. There were no quantitative alterations within the population of peripheral blood B or T lymphocytes in the studied cultures.

**Conclusion:**

Spinal manipulative treatment does not increase interleukin-2 -dependent polyclonal immunoglobulin synthesis by mitogen-activated B cells. However, antibody synthesis induced by interleukin-2 alone can be, at least temporarily, augmented following spinal manipulation. Thus, under certain physiological conditions spinal manipulative treatment might influence interleukin-2 -regulated biological responses.

## Background

The induction and regulation of immune responses involve complex interactions between the immune and nervous systems mediated by the biologic action of numerous humoral factors including neurotransmitters and immunoregulatory cytokines [1,2]. It has been suggested that systemic somatoautonomic reflex effects following spinal manipulative therapy (SMT) might include modulation of immune reactions [3,4]. Animal studies have found efferent sympathetic stimulation to be immunosuppressive [5] and it has been suggested that depressed levels of natural killer (NK) cells observed in low back patients [6] might be related to somatovisceral reflex stimulation. However, mechanisms of SMT action on immune modulation have remained illusive [7].

Demonstration of SMT-related effects on the production and/or biologic action of soluble regulators of the immune response provides a useful avenue for elucidating the immune consequences of SMT. Previous studies from our laboratory in asymptomatic subjects have demonstrated that a single high velocity low amplitude (HVLA) manipulation of the upper thoracic spine, characterized by cavitation and intended to mobilize a small joint fixation in the upper thoracic spine, has an inhibitory effect on proinflammatory cytokine production by peripheral blood mononuclear cells (PBMCs) [8]. Furthermore, in the same subjects, SMT with or without cavitation caused an enhancement of the *in vitro *capacity for mitogen-induced production of the immunoregulatory cytokine, interleukin-2 (IL-2) [9].

The above observations suggested that SMT-related biological effects might indeed include a range of quantitative/qualitative changes within the integrated cytokine network. However, it is not clear if or how such changes affect the response of immune effector cells. The present study addresses this issue by investigating whether SMT-related augmentation of the *in vitro *IL-2 synthesis by mitogen-activated T lymphocytes [9] coincides with the modulation of IL-2-dependent and/or IL-2 -induced responses of normal human B cells. To this end, *in vitro *antibody synthesis was determined in parallel PBMC cultures following stimulation with either pokeweed mitogen (PWM), which leads to T cell-mediated IL-2-dependent immunoglobulin (Ig) synthesis [10] or with exogenous human recombinant IL-2 (hrIL-2), which at sufficiently high concentration induces Ig synthesis by B cells [11].

## Methods

### Subjects

All subject-handling procedures were approved by the Canadian Memorial Chiropractic College Ethics Board. As indicated above, the present study represents a part of a larger investigation in which blood samples were obtained to test for changes in different parameters of the immune response following a spinal manipulative intervention [8,9]. In the present study, for determination of IL-2-dependent and IL-2- induced antibody production, samples were available from 74 of the subjects (Table 1).

**Table 1:** Demographic data of subjects.

Group	Age	Sex (F/M)
VC ( n = 22)	24.1 ± 1.5	14/8
SMT-NC ( n = 25)	25.3 ± 1.21	15/10
SMT-C ( n = 27)	24.8 ± 1.75	14/13

Results are presented as means ± SD.

Details of the experimental design and protocol have been described previously [8,9]. Briefly, subjects were accepted into the study if they had not received any manipulative treatments in the previous 6 months and the study clinician was able to identify a restricted motion segment in the upper thoracic spine (T1-T6). Subjects in whom no restrictions could be identified were dismissed from the study. Those accepted into the study were randomly assigned to one of 3 groups: spinal manipulation with cavitation (SMT-C), spinal manipulation without cavitation (SMT-NC) or venipuncture control (VC). SMT consisted of a single high velocity low amplitude adjustment in the form of a bilateral hypothenar push (Carver Bridge) [12], given on a single day, applied to the involved vertebral segment in a posterior-to-anterior direction, and with sufficient force, so as to produce joint cavitation as judged by the treating clinician. The procedure for SMT-NC consisted of an identical set-up using similar force but with positioning and line of drive altered to avoid cavitating the joint. In an earlier study using the same subjects, we had referred to this latter group as having received a sham manipulation [8]. Subjects in the VC group were treated similarly to the SMT-C and SMT-NC groups in every way except for the thrust.

### Blood samples

Peripheral blood was drawn in heparinized vacutainers (Becton Dickinson, Franklin Lakes, NJ) by venipuncture. Samples were collected prior to any manual intervention and then at 20 min and 2 hr post-treatment. A coding system was used in order to identify samples with a view of blinding the laboratory investigator(s) to the study groups. In every subject, samples collected before intervention served as a self-control (baseline) to which post-treatment responses were compared.

### Culture system

Peripheral blood mononuclear cells (PBMCs) were separated from heparinized blood samples by fractionation on Ficoll-Paque gradients (Pharmacia Biotech, Uppsala, Sweden). Cells collected from the interface were washed three times in RPMI 1640, enumerated and suspended in complete tissue culture medium (TCM) consisting of RPMI 1640 supplemented with 10% (v/v) fetal calf serum (pre-selected for low endotoxin level), 2 mM L-glutamine, 5 × 10^-5 ^M 2-mercaptoethanol (Sigma, St. Louis, MO) and antibiotics. To induce polyclonal antibody synthesis, duplicate PBMC cultures at a concentration of 0.5 × 10^6 ^cells/ml were stimulated, at initiation, with pokeweed mitogen (PWM, 10 μg/ml, Gibco, Grand Island, NY). Parallel preparations were stimulated with hrIL-2 derived from cDNA for human IL-2 in *E. coli *(Roche Diagnostics GmbH, Germany) at a final concentration of 200 U/culture according to the producer's specifications. Finally, inducer-free cultures were established in order to determine the level of spontaneous (background) synthesis of immunoglobulins (Igs) in each subject. All preparations were cultivated for 7 days in a humidified atmosphere of 5% CO2 and 95% air. At the end of incubation period, the culture supernatants were collected, aliquoted and were stored at -78°C. Samples were thawed immediately before testing and, to minimize inter-assay variability, all culture supernatants derived from a given subject were always examined in the same experiment.

### Phenotypic analyses of PBMCs

Enumeration of peripheral blood B and T lymphocytes in the preparations of PBMCs collected at baseline and then 2 hr post-treatment was carried out by flow cytometric analysis of samples following immunofluorescent staining with the respective anti-CD19 and anti-CD3 mouse anti-human monoclonal antibodies (BD Biosciences, Mississauga, ON).

### Assessment of immunoglobulin production

Polyclonal Ig synthesis was determined using the enzyme-linked immunosorbant assay (ELISA) technique essentially as described previously [13]. Briefly, duplicate dilutions of standards or culture supernatants in PBS-Tween were added to flat bottom microplate wells (Immulon 2HB, Thermo Labsystem, Franklin, MA) coated with anti-human immunoglobulin G (IgG) or immunoglobulin M (IgM) and incubated for 2 hr at 37°C. The plates were then washed thoroughly in PBS-Tween and incubated again (1 hr, 37°C) with a predetermined concentration of peroxidase-conjugated goat anti-human IgG or IgM. Following the development of color in the presence of a 0.4% solution of orthophenylenediamine (Sigma, ST. Lois, MO) and H_2_O_2_, the absorbance was measured at 492 nm using a Titertek Multiscan (Flow Laboratories, Helsinki, Finland). Concentrations of a given Ig were calculated from linearized (best fit) standard curves. Detection level for both IgG and IgM was 30 ng/ml. Each culture supernatant was tested at least 3 times and at several dilutions.

### Statistics

Levels of the induced IgG and IgM production in the study groups were evaluated for normality using the Shapiro-Francia test, and for equality of variances between groups using Levene's robust test statistic. For both outcome measures data were determined to be non-normal and equality of variances was not confirmed. As a result of these findings, the data was transformed prior to completing further analysis. The IgG data were transformed using the Box-Cox method, and thereafter were found to be normally distributed with equal variances between groups. The IgM data were transformed by taking the natural log of each value, and thereafter were also found to be normally distributed with equal variances between groups. Statistical significance of differences between the study groups (V C, SMT-NC, SMT-C) and within groups (baseline vs. post-treatment at 20 min vs. post-treatment at 2 h) was then determined using the repeated measures ANOVA. This was followed by post-hoc Tukey's HSD test for pairwise comparisons at each time point [14]. Statistical significance was accepted at p < 0.05. Data were analyzed using STATA SE 8 Sofware.

## Results

### Cell enumeration

A single SMT had no effect on the overall number of PBMCs compared to both baseline and venipuncture controls. Also, at two hours post-treatment, the number of lymphocytes expressing the CD19 or CD3 phenotypes (B and T cells respectively) remained unchanged in all study groups (Table 2). Thus, the cellular compositions of cultures derived from blood samples in the VC, SMT-NC and SMT-C subjects were comparable.

**Table 2:** The proportion of B (CD19) and T (CD3) lymphocytes within the population of peripheral blood mononuclear cells from subjects studied prior to (baseline), and 2 hr after treatment. Results are presented as means ± SD.

Group	Phenotype [%]			
	CD 19 (range)		CD3 (range)	
	Baseline	2 hr	Baseline	2 hr
VC	9.2 ± 3 (6 - 14)	10.6 ± 4 (6 - 16)	77 ± 12 (67 - 90)	76 ± 11 (68 - 88)
SMT-NC	10.8 ± 3 (6 - 11)	11.8 ± 5 (7 - 17)	74 ± 14 (64 - 89)	71 ± 16 (58 - 84)
SMT-C	11.0 ± 4 (7 - 15)	11.4 ± 6 (6 - 17)	72 ± 15 (62 - 88)	75 ± 12 (64 - 94)

### PWM-induced IgG and IgM production

**Figure 1:**
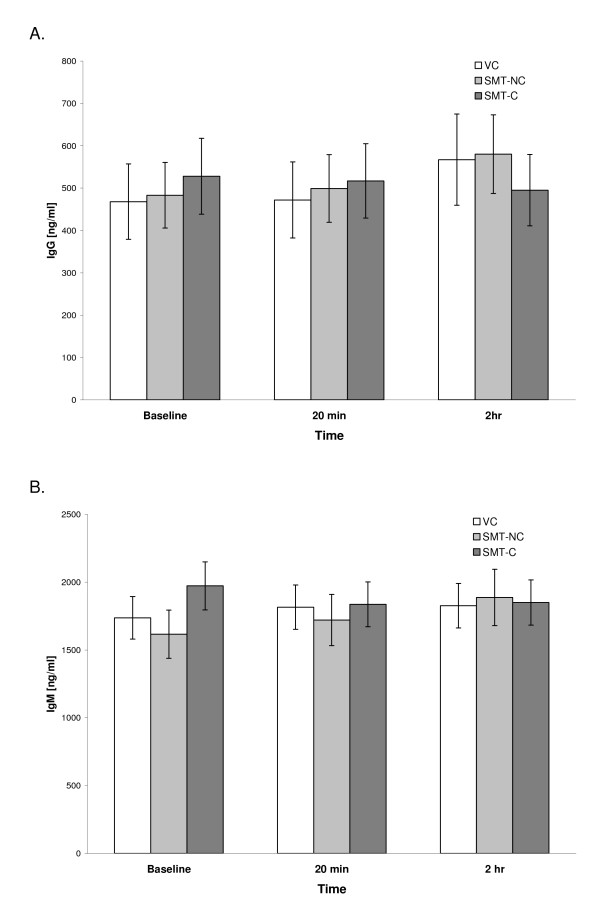
**Effect of SMT on the *in vitro *production of IgG (A) and IgM (B) induced by PWM stimulation of PBMCs**. Cultures were prepared from blood samples collected from the venipuncture control (VC) and experimental (SMT-NC, SMT-C) groups at indicated time points and activated with pokeweed mitogen (PWM, 10 μg/ml) at initiation. Concentrations of newly synthesized IgG in culture supernatants were determined after 7 days of cultivation by a specific immunoassay. The values depict untransformed means ± SEM of immunoglobulin synthesis for each of the study groups.

In the majority of cultures, the background (spontaneous) secretion of Igs in inducer-free cultures did not exceed 100 ng/ml or remained below the level of detection. On the other hand, stimulation of parallel cultures with PWM induced the synthesis of both IgG and IgM classes of antibodies in all of the studied preparations. Figure 1A and 1B illustrate the levels of both Igs produced in PWM-stimulated PBMC cultures set up prior to the treatment (baseline) and then at 20 min and 2 hr post-intervention.

The baseline quantities of IgG and IgM synthesized by the subjects were comparable across the study groups. Over the 2 hr of the study period, the mean production of both IgG and IgM in cultures from VC, SMT-NC and SMT-C manipulated subjects was essentially unaltered and remained within the range of the normal human *in vitro *response generated following PWM stimulation [13] (Figure 1A and 1B).

### IL-2-induced IgG production

Due to insufficient numbers of PBMCs in fractionated blood preparations from 11 subjects (3 from VC, 4 from SMT-NC and 4 from SMT-C groups), studies were completed in 63/74 enrolled individuals. As expected, the production of IL-2-induced Igs was considerably lower, in all cultures, compared to that induced by PWM [15].

**Figure 2:**
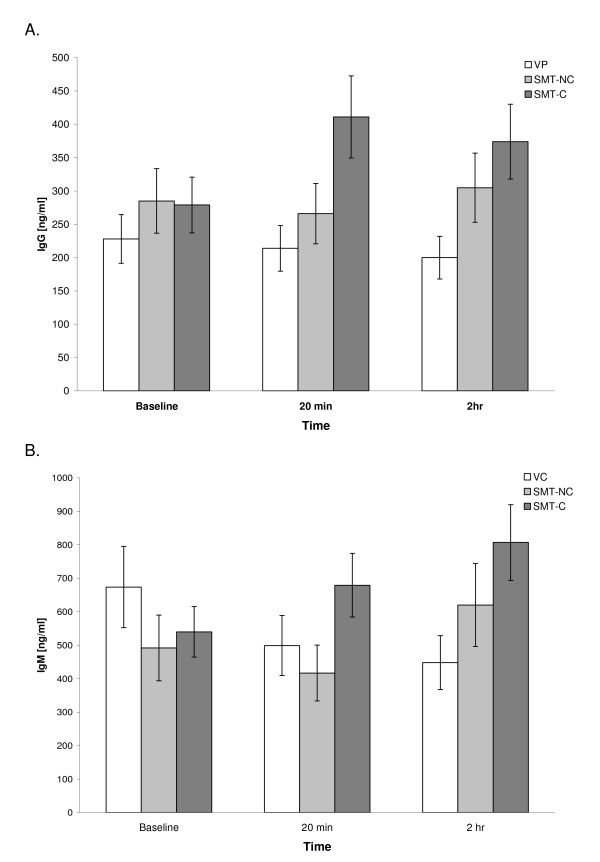
**Effect of SMT on IL-2- induced IgG (A) and IgM (B) production in PBMC cultures**. Cultures established at the indicated time intervals after the treatment were activated at initiation with human recombinant IL-2 (200 U/ml). The levels of immunoglobulin in supernatants collected after 7 days of cultivation were determined by a specific immunoassay. The results are presented as untransformed means of values ± SEM for each of the study groups.

Figure 2A depicts the means of IL-2-induced IgG synthesis in PBMC cultures from the studied subjects. The repeated measures ANOVA of the transformed data demonstrated a statistically significant group by time interaction effect (F = 2.8, P = 0.03) with respect to IgG production. Post-hoc Tukey's HSD pairwise comparisons between the study groups demonstrated that no significant differences existed at baseline. However, at 20 min post-treatment, the mean production of IgG in the SMT-C group was significantly higher than that in the VC and SMT-NC groups. At 2 hr post-treatment, the production of IgG in cultures from both SMT-C and SMT-NC was significantly elevated compared to VC.

### IL-2-induced IgM production

Figure 2B illustrates post-treatment alterations in the mean level of IgM synthesis in all groups. The repeated measures ANOVA of the transformed IgM data also indicated a statistically significant (F = 2.68, P = 0.04) group by time interaction effect. Post-hoc Tukey's HSD pairwise comparisons determined that, at 2 hr post-treatment, the mean level of IgM synthesis in the SMT-C group was significantly elevated compared with the VC group (Figure 2B).

## Discussion

Results of the present investigation demonstrate that in normal asymptomatic subjects in whom a restricted upper thoracic motion segment was identified, neither venipuncture alone nor a single spinal manipulation with or without cavitation affected the capacity for the IL-2 -dependent (i.e. T-cell-dependent), PWM-triggered antibody production examined within 2 hr post-intervention. However, within the same time frame, antibody synthesis (both IgG and IgM class) *induced *by hrIL-2 was significantly augmented in cultures from subjects treated with SMT-C.

The mechanism(s) underlying the significant amplification of the response to exogenous IL-2 in SMT-C treated subjects is unknown. The possibility that the observed effect was related to an increase in the total content of IL-2 in these cultures cannot be excluded. The IL-2-inducible immunoglobulin synthesis is a dose-dependent process and requires high concentrations of this cytokine [16]. As reported previously, the intrinsic capacity for IL-2 production in cultures from SMT-treated subjects is enhanced [9]. Considering the fact that IL-2 up-regulates its own production, as well as the expression of specific IL-2 receptors [17,18], it is feasible that the production of endogenous IL-2 was indeed up-regulated in the presence of hrIL-2, and more so in subjects treated with SMT-C. Furthermore, the increase in the total IL-2 level could facilitate the release of other soluble mediators of the humoral immune response by functional T cells present in the studied cultures and subsequently enhance antibody secretion by B cells [19]. Noteworthy, a significant increase in the level of IgG production was observed also, at 2 hr post-treatment, in subjects who received SMT-NC manipulation (Figure 2A). This is consistent with our earlier findings of the time-limited effect of SMT-NC on T lymphocytes [9].

The above considerations notwithstanding, it is doubtful that the combined action of endogenous and exogenous IL-2 could be the sole mechanism of the observed up-regulation of IL-2-induced Ig synthesis in the SMT-C group. Normal human B cells express functional (high affinity) IL-2 receptors (IL-2R) and thus IL-2 plays a significant role in the modulation of B cell function [20]. Therefore, it is feasible that following SMT-C, the interaction between IL-2 and its specific high affinity receptor (IL-2R) on the surface of B lymphocytes was somewhat facilitated and resulted in augmentation of Ig synthesis. However, the effect of SMT-C on the capacity of B lymphocytes for the expression of IL-2R was not investigated in this study.

It is also possible that the increase in IL-2 induced antibody production in SMT-C treated subjects was related, directly or indirectly, to the biologic action(s) of other soluble mediators released as a consequence of spinal manipulation. The cross- talk between the soluble mediators produced by the immune and nervous systems regulates the magnitude and duration of both immune and inflammatory responses [21,22]. Indeed, the observation of attenuated production of proinflammatory cytokines in subjects treated with SMT-C [8] prompted our exploratory studies on potential mechanisms of this effect. Studies still in progress in this laboratory indicate that PWM-activated cultures from SMT-C -treated, but not SMT-NC or VC subjects contain significantly elevated levels of the anti-inflammatory cytokine interleukin 10 (IL-10) [23]. IL- 10 has been shown to increase the affinity of the B cell receptor for IL-2 resulting in a putative improvement of signal transduction and promotion of B lymphocyte activation [24]. Furthermore, IL-10 synergizes with the available IL-2 to increase synthesis of Igs but has no effect on T-cell dependent polyclonal responses [25-27]. In the present study PWM-induced, T-cell dependent antibody synthesis was indeed not altered following SMT (Figure 1). Thus, it is feasible that IL-2-induced IgG and IgM production, in cultures obtained from SMT-C treated subjects (Figure 2), was augmented due to enhancement of IL-2 signalling by endogenous IL-10.

The suggested facilitation of Ig synthesis due to SMT may be associated with joint cavitation. However, in this regard the design of our experiments did not control or measure the actual forces delivered during the manipulative procedure. Although the intention was to deliver a manipulative thrust of similar force (but different direction) for both the cavitation and no cavitation groups, the forces delivered to the no cavitation group may have been smaller. We have previously discussed the issue of cavitation in the context of the effects of manipulation [9].

The clinical significance of the elevated responsiveness to IL-2 demonstrated in this *in vitro *study is presently unclear. It should be noted that augmentation of IL-2-induced IgG or IgM synthesis in the SMT-C group, although statistically significant, did not exceed the physiological range of normal human response [13,28]. Nonetheless, results of the present pilot study provide the first experimental evidence that systemic sequelae of spinal manipulative therapy include functional changes in the ability of peripheral blood lymphocytes to respond to immunoregulatory mediators and the clinical relevance of such alterations should be further explored.

## Conclusion

In the *in vitro *model utilizing PBMC cultures derived from asymptomatic subjects receiving a spinal manipulative intervention, or undergoing venipuncture procedure alone, immunoglobulin synthesis is augmented by manipulation. The mechanism mediating this process appears to involve direct activation of B cells by exogenous IL-2 rather than T-cell dependent interactions. The results suggest that the systemic consequences of SMT may encompass a "priming" effect on the immune effector cells thereby altering their response to certain immunoregulatory mediators.

## List of Acronyms

ELISA - Enzyme linked immunosorbant assay, hrIL-2 - Human recombinant interleukin 2, HVLA - High velocity low amplitude, Ig - Immunoglobulin, IgG - Immunoglobulin G, IgM - Immunoglobulin M, IL-2 - Interleukin 2, IL-2R - Interleukin 2 receptor, IL-10 - Interleukin 10, PBMC - Peripheral blood mononuclear cell, PBS - Phosphate buffered saline, SMT - Spinal manipulative treatment (or therapy), SMT-C - Spinal manipulative treatment associated with cavitation, SMT-NC - Spinal manipulative treatment without cavitation, VC - Venipuncture control

## Competing interests

The authors declare that they have no competing interests.

## Authors' contributions

JTI contributed to the design of the study, was responsible for all laboratory procedures, analysis of data, and contributed to the writing of the manuscript. MM performed statistical analysis and contributed to writing of the manuscript. HSI contributed to the design of the study, was responsible for subject recruitment and coordination of the study, analysis of data, and contributed to the writing of the manuscript. RR contributed to the design of the study and was the study clinician. All authors have read and approved the final manuscript.
